# Allogeneic stem cell transplantation mitigates the adverse prognostic impact of high diagnostic *BAALC* and *MN1* expression in AML

**DOI:** 10.1007/s00277-020-04235-8

**Published:** 2020-08-29

**Authors:** Madlen Jentzsch, Marius Bill, Juliane Grimm, Dominic Brauer, Donata Backhaus, Karoline Goldmann, Julia Schulz, Dietger Niederwieser, Uwe Platzbecker, Sebastian Schwind

**Affiliations:** Medical Clinic and Policlinic 1, Hematology and Cellular Therapy, Leipzig University Hospital, Liebigstraße 22, Haus 7, 04103 Leipzig, Germany

**Keywords:** Acute myeloid leukemia, *BAALC*, *MN1*, Allogeneic stem cell transplantation

## Abstract

For most acute myeloid leukemia (AML) patients, an allogeneic hematopoietic stem cell transplantation (HSCT) offers the highest chance of sustained remissions and long-term survival. At diagnosis, high expression of the AML-associated genes *BAALC* (brain and acute leukemia, cytoplasmic) and *MN1* (meningioma-1) were repeatedly linked to inferior outcomes in patients consolidated with chemotherapy while data for patients receiving HSCT remain limited. Using clinically applicable digital droplet PCR assays, we analyzed the diagnostic *BAALC/ABL1* and *MN1/ABL1* copy numbers in 302 AML patients. High *BAALC/ABL1* and *MN1/ABL1* copy numbers associated with common adverse prognostic factors at diagnosis. However, while high diagnostic copy numbers of both genes associated with shorter event free survival (EFS) and overall survival (OS) in patients receiving chemotherapy, there was no prognostic impact in patients undergoing HSCT. Our data suggests that the adverse prognostic impact of high *BAALC* and *MN1* expression are mitigated by allogeneic HSCT. But preHSCT *BAALC*/*ABL1* and *MN1*/*ABL1* assessed in remission prior to HSCT remained prognosticators for EFS and OS independent of the diagnostic expression status. Whether allogeneic HSCT may improve survival for AML patients with high diagnostic *BAALC* or *MN1* expression should be investigated prospectively and may improve informed decisions towards individualized consolidation options in AML.

**Electronic supplementary material:**

The online version of this article (10.1007/s00277-020-04235-8) contains supplementary material, which is available to authorized users.

## Introduction

Acute myeloid leukemia (AML) is a highly heterogeneous disease for which reliable risk stratifications are needed to individualize treatment strategies [1]. Today, potential consolidation therapies for AML patients in remission after successful induction therapy include intensive chemotherapy cycles alone or an allogeneic hematopoietic stem cell transplantation (HSCT). Through immunological graft-versus-leukemia (GvL) effects, where the donor’s immunocompetent cells are believed to eradicate residual disease [2, 3], allogeneic HSCT remains the treatment option with the highest chance of sustained remissions in most AML patients, albeit the associated morbidity and mortality [1].

The AML-associated genes *BAALC* (brain and acute leukemia, cytoplasmatic) and *MN1* (meningioma-1) have been shown to be physiologically expressed at high levels in myeloid progenitor cells and downregulated during maturation and to promote leukemogenesis through blockage of myeloid differentiation [4–6]. While *BAALC* maps to chromosome band 8q22.3 and was initially identified in AML patients harboring a trisomy 8 [7], *MN1* is located on chromosome 22q12.3 and a transcription coactivator firstly described in meningioma pathogenesis [8]. High expression levels of both genes at AML diagnosis have repeatedly been associated with adverse outcomes in both younger [4, 9] and older AML patients [10, 11], especially in the context of a normal karyotype [12–14]. Furthermore, the expression levels of both genes have been identified as feasible markers for residual disease in AML patients in complete remission (CR) independent of the applied consolidation therapy [15–19].

However, the majority of the studies investigating the prognostic impact of diagnostic *BAALC* and *MN1* expression levels focused on patients consolidated with standard cytarabine-based chemotherapies or autologous HSCT in which either none or only a small number of the analyzed individuals received allogeneic HSCT for consolidation. Only one recently published manuscript analyzed the data of 71 AML patients from The Cancer Genome Atlas (TCGA) and suggested no prognostic impact of *BAALC* expression levels at diagnosis in patients receiving allogeneic HSCT [20]. This study was restricted by patient numbers and limited information on the applied treatments (e.g., intensity of conditioning regimens). Here, we analyzed the prognostic significance of the differential diagnostic *BAALC* and *MN1* expression levels in a well-defined cohort of AML patients who were either treated with chemotherapy alone or received an allogeneic HSCT as consolidation therapy at our institution. For better reproducibility, and to develop a feasible clinical routine assay, we adopted a digital droplet PCR (ddPCR) technology for absolute diagnostic *BAALC* and *MN1* quantification [21].

## Subjects and methods

### Patients and treatment

We analyzed the diagnostic bone marrow material of 302 AML patients who were treated at the University of Leipzig between November 2000 and October 2018 for their *BAALC*/*ABL1* and *MN1*/*ABL1* copy numbers. Median age at diagnosis was 62.2 years (range 14.5–87.8 years). All nonAPL karyotypes were included in the analysis. Written informed consent was obtained from all patients in accordance with the Declaration of Helsinki. For all 302 patients, associations of diagnostic *BAALC*/*ABL1* and *MN1*/*ABL1* copy numbers with baseline clinical and genetic factors were assessed (“association set”). Of the 207 patients who received an allogeneic HSCT for consolidation therapy, 186 patients were transplanted in CR or CR with incomplete peripheral recovery (CRi) and were eligible for outcome analyses. Of the 95 patients who were treated with chemotherapy alone, 77 patients received at least one cycle of intensive chemotherapy and survived 28 days after diagnosis and were also included in the outcome analyses. Thus, outcome was evaluated for 263 AML patients (“outcome set”). For details, please see the flow chart in Supplementary Fig. S1.

All patients in the outcome set received age-dependent standard cytarabine–based chemotherapy protocols (please see Supplementary Information for details). Conditioning regimens in the 186 patients receiving allogeneic HSCT were either myeloablative (*n* = 47, using 2 × 60 mg/kg body weight cyclophosphamide and 12 Gray [Gy] total body irradiation) or nonmyeloablative (*n* = 139, using 3 × 30 mg/m^2^ fludarabine and 2 Gy total body irradiation). Median time from diagnosis to allogeneic HSCT was 139 days. Reasons for the chosen consolidation therapy as well as conditioning regime in case of allogeneic HSCT are given in the Supplementary Information. All transplanted patients received granulocyte colony stimulating factor–stimulated peripheral blood stem cells. Stem cell donors were human leukocyte antigen (HLA) matched related (*n* = 42, 23%), HLA matched unrelated (*n* = 108, 58%) or HLA mismatched unrelated (*n* = 36, 19%). Further patients’ characteristics are shown in Table 1 and Supplementary Table S1 and S2. Median follow-up after diagnosis was 5.0 years for patients alive.

**Table 1:** Clinical and genetic characteristics for all patients according to *BAALC/ABL1* (high vs low, median cut) and *MN1/ABL1* (high vs low, median cut) copy numbers at diagnosis (*n* = 302)

	All patients *n* = 302	low *BAALC/ABL1* copy numbers *n* = 151	high *BAALC/ABL1* copy numbers *n* = 151	*P*	low *MN1/ABL1* copy numbers *n* = 151	high *MN1/ABL1* copy numbers *n* = 151	*P*
Clinical information at diagnosis							
Age at diagnosis, years				0.55			0.74
Median Range	62.2 14.5–87.8	63.2 14.5–87.8	61.5 19.5–82.7		62.7 14.5–87.8	61.9 19.5–82.7	
Sex, *n* (%)				0.82			0.49
Male Female	147 155	75 (50) 76 (50)	72 (48) 79 (52)		77 (51) 74 (49)	70 (46) 81 (54)	
Disease origin, *n* (%)				0.08			0.13
Secondary De novo	92 210	39 (26) 112 (74)	53 (35) 98 (65)		40 (26) 111 (74)	52 (37) 99 (63)	
Hemoglobin, g/dL				0.42			0.22
Median Range	8.9 4.3–14.9	8.8 4.3–14.4	8.7 5.0–14.9		8.9 4.3–14.4	8.8 5.0–14.9	
Platelet count, × 10^9^/L				0.27			0.44
Median Range	63 2–391	65 2–305	51 2–391		63 3–305	53 2–391	
WBC, × 10^9^/L				< 0.001			< 0.001
Median Range	6.5 0.6–385	18.7 0.6–385	6.5 0.7–295		18.8 0.6–385	6.4 0.7–295	
Blood blasts, %				0.88			0.32
Median Range	25 0–98	22 0–97	27 0–98		28 0–97	24 0–98	
BM blasts, %				0.48			0.65
Median Range	55 3–98	50 3–98	55 10–95		52 3–98	55 0–95	
BM CD34 expression, %				< 0.001			< 0.001
Median Range	31 0–97	1.7 0–83	50 0–97		2 0–90	48 0–97	
BM CD34+/CD38- burden, %				< 0.001			< 0.001
Median Range	0.5 0–89	0.1 0–60	1 0–89		0.1 0–75	1 0–89	
Genetic information at diagnosis							
Normal karyotype, *n* (%)				< 0.001			< 0.001
Absent Present	160 129	50 (35) 91 (65)	110 (74) 38 (26)		58 (41) 83 (59)	102 (69) 46 (31)	
ELN2017 group, *n* (%)				< 0.001			< 0.001
Favorable Intermediate Adverse	94 71 101	65 (49) 31 (23) 38 (28)	29 (22) 40 (30) 63 (48)		67 (42) 26 (15) 39 (43)	27 (51) 45 (20) 62 (30)	
*NPM1*, *n* (%)				< 0.001			< 0.001
Wild-type Mutated	214 83	72 (49) 76 (51)	142 (95) 7 (5)		71 (48) 78 (52)	143 (97) 5 (3)	
*CEBPA*, *n* (%)				0.29			0.006
Wild-type Mutated	206 25	109 (92) 10 (8)	97 (87) 15 (13)		111 (95) 6 (5)	95 (83) 19 (17)	
*FLT3*-ITD, *n* (%)				0.001			0.004
Absent Present	243 54	110 (74) 38 (26)	133 (89) 16 (11)		112 (75) 37 (25)	131 (89) 17 (11)	
*RUNX1*, *n* (%)				0.004			0.004
Wild-type Mutated	78 11	44 (98) 1 (2)	34 (77) 10 (23)		44 (98) 1 (2)	34 (77) 10 (23)	
*ASXL1*, *n* (%)				1			0.26
Wild-type Mutated	75 14	38 (84) 7 (16)	37 (84) 7 (16)		40 (89) 5 (11)	35 (80) 9 (20)	
*TP53*, *n* (%)				0.49			0.49
Wild-type Mutated	80 9	39 (87) 6 (13)	41 (93) 3 (7)		39 (87) 6 (13)	41 (93) 3 (7)	
*BAALC* copy numbers at diagnosis, *n* (%)							< 0.001
Low High	34 102	–	–	–	133 (88) 18 (12)	18 (12) 133 (88)	
*MN1* copy numbers at diagnosis, *n* (%)				< 0.001			
Low High	151 151	133 (88) 18 (12)	18 (12) 133 (88)		–	–	–

*ASXL1*, Additional sex combs-like 1 gene; *BM*, bone marrow; *BAALC*, brain and acute leukemia cytogenetic gene; *CEBPA*, CCAAT/enhancer-binding protein alpha gene; *ELN*, European LeukemiaNet; *FLT3-ITD*, internal tandem duplication of the FLT3 gene; *Hb*, hemoglobin; *MN1*, meningioma 1 gene; *NPM1*, nucleophosmin 1 gene; *PB*, peripheral blood; *RUNX1*, Runt-related transcription factor 1 gene; *TP53*, tumor protein 53 gene; *WBC*, white blood count

### Assessment of *BAALC*/*ABL1* and *MN1*/*ABL1* copy numbers and cutoff point definitions

For all patients, absolute *BAALC* and *MN1* copy numbers at diagnosis were assessed using specific ddPCR assay (BioRad, Hercules, California, USA). ddPCR was performed on a QX100 platform (BioRad), and QuantaSoft software (Biorad) was used for raw data processing as previously described [15]. Both genes were normalized to *ABL1* copy numbers as internal control. To evaluate the prognostic impact, the median *BAALC*/*ABL1* (absolute 0.2538) and *MN1*/*ABL1* copy numbers (absolute 0.2424) were used to define patients with high or low *BAALC*/*ABL1* and *MN1*/*ABL1* copy numbers at diagnosis. For validation of the ddPCR results, in 110 patients, qRT-PCR was performed to assess *BAALC* and *MN1* expression levels at diagnosis additionally to ddPCR. For details regarding qRT-PCR analysis, please see Supplementary Information.

We previously reported on the prognostic significance of preHSCT *BAALC* [15] as well as preHSCT *MN1* copy numbers [16]. In the here-presented patient population, preHSCT *BAALC*/*ABL1* and preHSCT *MN1*/*ABL1* copy numbers were available in 77 and 76 patients, respectively. The previously published cutoffs were used to define patients with high or low preHSCT *BAALC/ABL1* and preHSCT *MN1/ABL1* copy numbers [15, 16].

### Cytogenetics, molecular marker, and flow cytometry

Diagnostic cytogenetic analyses were performed centrally using standard techniques of banding and in situ hybridization. Bone marrow mononuclear cells at diagnosis were assessed for surface presence of an institutional standard panel as previously described [22]. The mutation status of the CCAAT/enhancer-binding protein alpha (*CEBPA*), nucleophosmin 1 (*NPM1*), and *FLT3* tyrosine kinase (*FLT3*-TKD) gene as well as the presence or absence of internal tandem duplications in the *FLT3* gene (*FLT3*-ITD) were evaluated as previously described [23]. For patients with material available, mutation status of 54 genes included in the TruSight Myeloid Sequencing Panel (Illumina) was evaluated at diagnosis as previously described [22, 24]. Patients were grouped according to the ELN2017 genetic classification [1].

### Definition of clinical endpoints and statistical analyses

All statistical analyses were performed using the R statistical software platform (version 3.4.3) [25]. Overall survival (OS) was calculated from diagnosis until death from any cause. Event free survival (EFS) was calculated from diagnosis to event (i.e., nonachievement of a CR or CRi after two cycles of chemotherapy, relapse or death from any cause). Associations with baseline clinical, demographic, and molecular features were compared using the Kruskal–Wallis Test and Fisher’s exact tests for continuous and categorical variables, respectively. Survival estimates were calculated using the Kaplan–Meier method, and groups were compared using the log-rank test. Multivariate analyses methods are described in the Supplementary Information.

## Results

### Comparison of qRT-PCR and ddPCR results

**Fig. 1:**
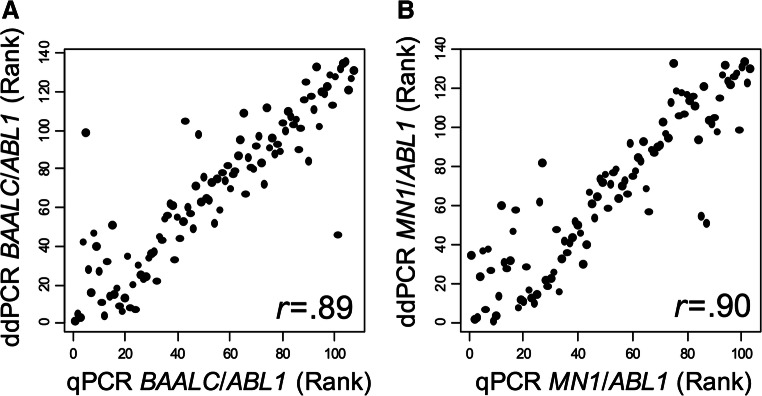
Spearman correlation between ddPCR and qRT-PCR at diagnosis **a** *BAALC/ABL1* and **b** *MN1/ABL1*

To validate our ddPCR-based expression assays, we compared the results to classical qRT-PCR assays. Results from gene expression analysis by qRT-PCR and copy number analysis by ddPCR correlated well (Spearman correlation coefficient: *BAALC r* = 0.89 and *MN1 r* = 0.90, Fig. 1).

### Associations *of BAALC*/*ABL1* copy numbers at diagnosis with clinical and genetic characteristics

Patients with high *BAALC/ABL1* copy numbers at diagnosis had a lower white blood count at diagnosis (*P* < .001) and presented with a higher expression of immature surface antigens (i.e., CD34, *P* < 0.001; CD34+/CD38−, *P <* 0.001; and CD117, *P* < 0.001), higher expression of surface antigens indicating T cell differentiation (i.e., CD7, *P* < 0.001; and CD2, *P* < 0.001), higher CD13 expression (*P* = 0.04), but lower expression of other antigens indicating myeloid differentiation (i.e., CD64, *P* < 0.001; CD11b, *P* = 0.01; and CD33, *P* = 0.001) on mononuclear bone marrow cells at diagnosis (Supplementary Table S1). They had a lower frequency of a normal karyotype (*P* < 0.001) and were more likely to have a core binding factor AML (CBF-AML, *P* < 0.001) but also to harbor adverse-risk genetics, i.e. del(5)/del(5q) (*P* = 0.001), del(7)/del(7q) (*P* = 0.001), a monosomal karyotype (*P* = 0.02) [26], a complex karyotype (*P* = 0.02) [1], as well as worse risk according to ELN2017 classification (*P* < 0.001, Table 1). High *BAALC*/*ABL1* copy numbers also associated with a lower frequency of *NPM1* mutations (*P* < 0.001), *FLT3*-ITD (*P* < 0.001), *DNMT3A* mutations (*P* = 0.03), by trend *TET2* mutations (*P* = 0.10), and a higher frequency of *RUNX1* mutations (*P* = 0.004), higher *MN1/ABL1* copy numbers (*P* < 0.001), higher *GPR56* expression (*P* < 0.001), and by trend higher *EVI1* expression (*P* = 0.08) at diagnosis.

### Associations *of MN1*/*ABL1* copy numbers at diagnosis with clinical and genetic characteristics

Patients with high *MN1*/*ABL1* copy numbers at diagnosis had lower white blood count at diagnosis (*P* < 0.001) and presented with a higher expression of immature surface antigens (i.e., CD34, *P <* 0.001; CD34+/CD38−, *P* < 0.001; and CD117, *P* < 0.001), higher expression of surface antigens indicating T cell differentiation (i.e., CD2, *P* < 0.001 and CD7, *P* < 0.001), higher CD13 (*P* = 0.007), but lower expression of other antigens indicating myeloid differentiation (i.e., CD33, *P* < 0.001; CD15, *P* = 0.05; and CD64, *P* = 0.001) on mononuclear bone marrow cells at diagnosis (Supplementary Table S1). High *MN1*/*ABL1* copy numbers also associated with a lower frequency of a normal karyotype (*P* < 0.001) and a higher frequency of CBF-AML (*P* = 0.001) but also a higher frequency of adverse risk genetics as del(7)/del(7q) (*P* = 0.001), del(5)/del(5q) (*P* = 0.01), by trend monosomal karyotype (*P* = 0.09) and worse risk according to ELN2017 classification (*P* < 0.001, Table 1). High *MN1*/*ABL1* copy numbers also associated with a lower frequency of *NPM1* mutations (*P* < 0.001), *FLT3*-ITD (*P* = 0.004), *CEBPA* mutations (*P* = 0.006), by trend *TET2* mutations (*P* = 0.10), as well as a higher frequency of *RUNX1* mutations (*P* = 0.004), higher *BAALC*/*ABL1* copy numbers (*P* < 0.001), and higher *GPR56* expression (*P* < 0.001) at diagnosis.

### Prognostic impact of *BAALC*/*ABL1* and *MN1*/*ABL1* copy numbers at diagnosis

**Fig. 2:**
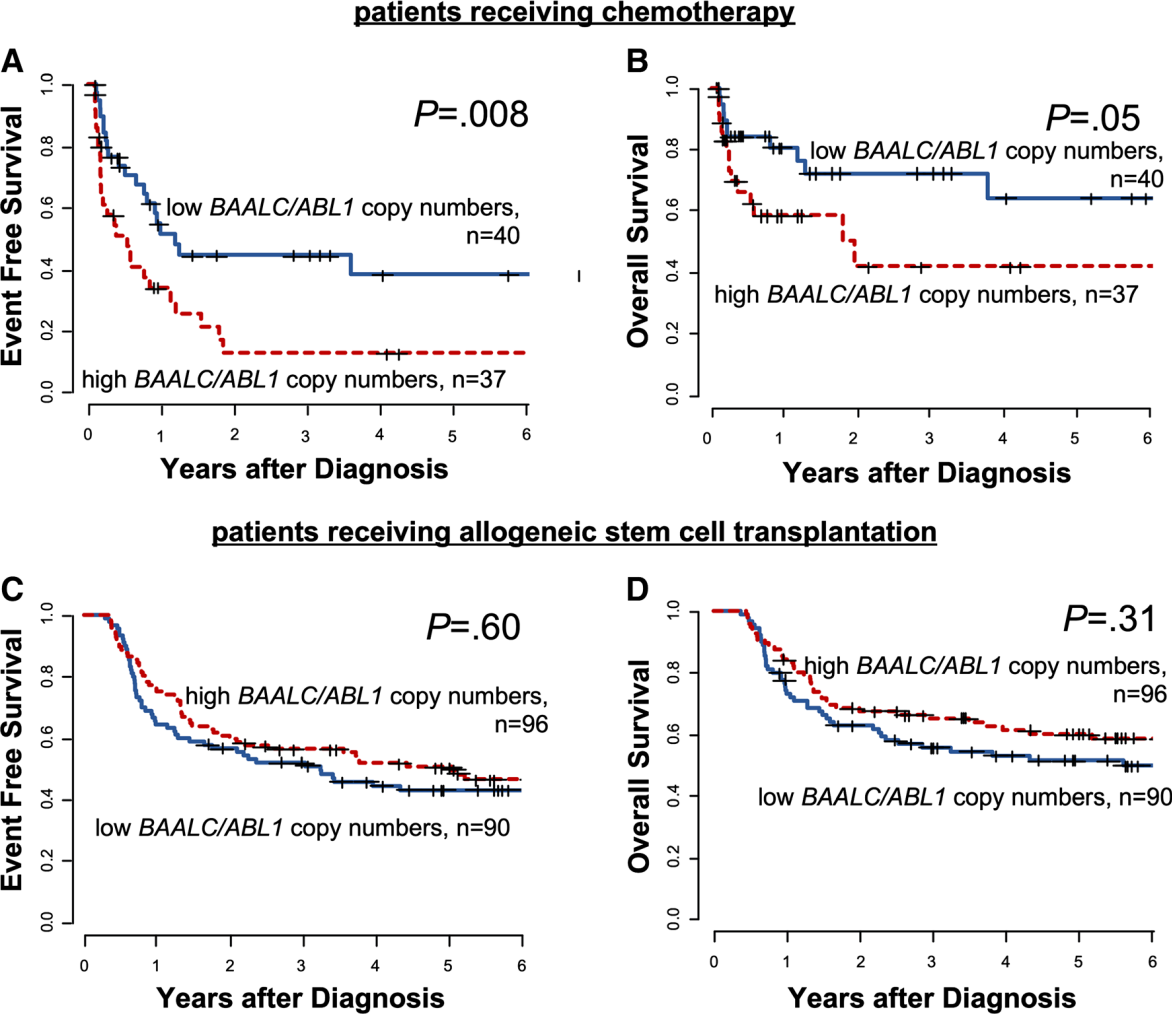
Outcome according to *BAALC/ABL1* at diagnosis in AML patients (“outcome set”, *n* = 263). **a** Event free survival and **b** overall survival according in patients receiving chemotherapy alone and **c** event free survival and **d** overall survival in patients consolidated with an allogeneic stem cell transplantation in CR/CRi

In line with previously published reports, in patients treated with chemotherapy alone, *BAALC*/*ABL1* copy numbers at diagnosis associated with a significantly shorter EFS (*P* = 0.008, Fig. 2a) as well as shorter OS (*P* = 0.05, Fig. 2b). In contrast, in patients receiving allogeneic HSCT as consolidation therapy, there was no different EFS (*P* = 0.60, Fig. 2c) or OS (*P* = 0.31, Fig. 2d) in patients with high or low *BAALC*/*ABL1* copy numbers at diagnosis.

**Fig. 3:**
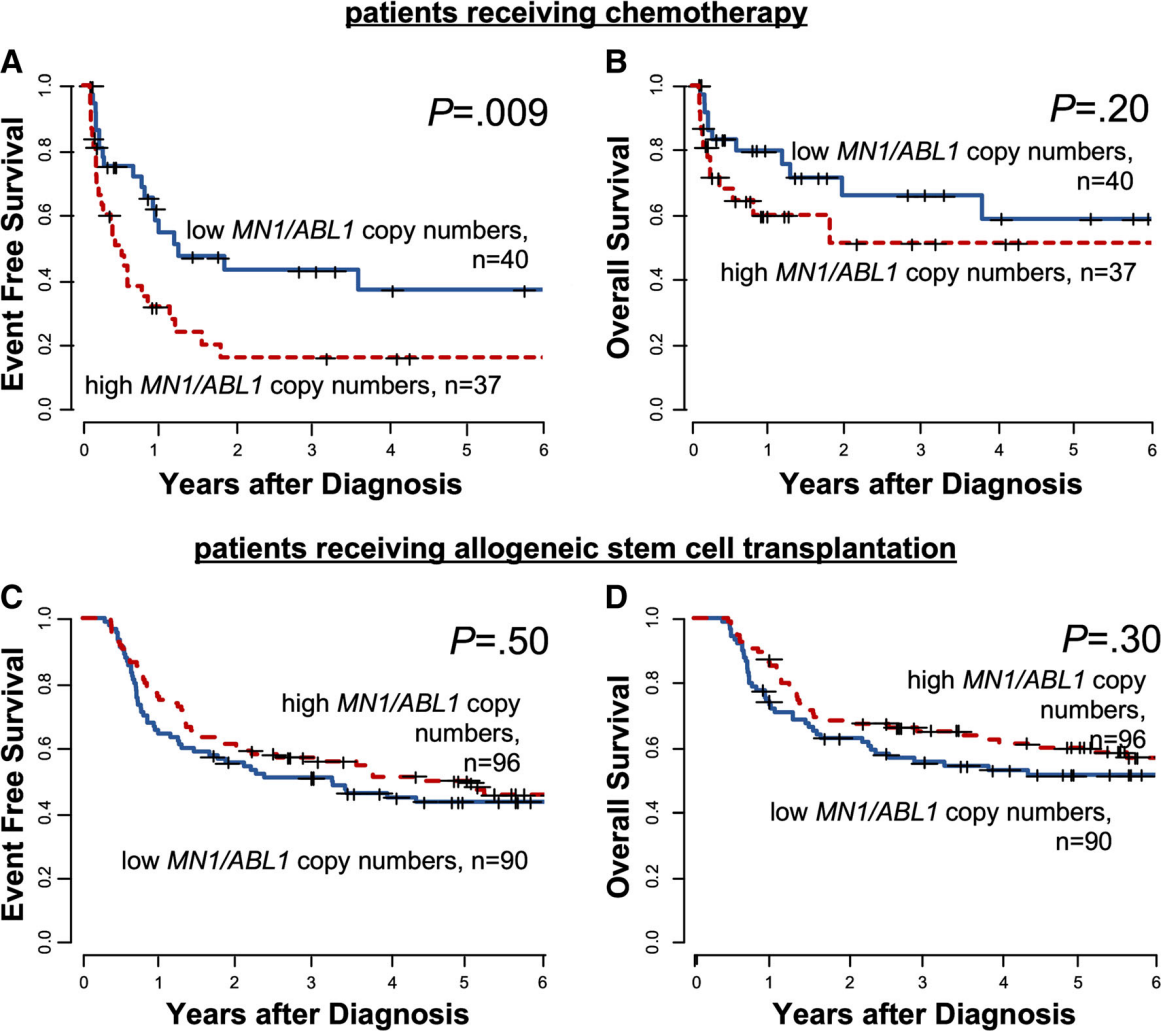
Outcome according to *MN1/ABL1* at diagnosis in AML patients (“outcome set”, n = 263). **a** event free survival and **b** overall survival according in patients receiving chemotherapy alone and **c** event free survival and **d** overall survival in patients consolidated with an allogeneic stem cell transplantation in CR/CRi

Similarly, high *MN1*/*ABL1* copy numbers associated with shorter EFS (*P* = 0.009, Fig. 3a), which despite a separation of the curves did not translate into significantly shorter OS (*P* = 0.20, Fig. 3b). Again, in patients receiving allogeneic HSCT as consolidation therapy, there was no different EFS (*P* = 0.50, Fig. 3c) or OS (*P* = 0.30, Fig. 3d) in patients with high or low *MN1*/*ABL1* copy numbers at diagnosis.

In multivariate analyses for patients consolidated with chemotherapy, high *MN1*/*ABL1* copy numbers at diagnosis remained a significant factor for shorter EFS after adjustment for age at diagnosis and presence of a monosomal karyotype while high *BAALC*/*ABL1* copy numbers at diagnosis remained a significant factor shorter OS after adjustment for hemoglobin levels at diagnosis and presence of a complex karyotype (Table 2). Also in multivariate analyses neither high *BAALC*/*ABL1* nor high *MN1/ABL1* copy numbers at diagnosis were significantly associated with EFS or OS in patients receiving allogeneic HSCT (Table 3).

**Table 2:** Multivariate analysis for patients in the outcome set receiving chemotherapy (*n* = 77)

	Event free survival		Overall survival	
	HR* (95% CI)	*P*	OR** (95% CI)	*P*
Age at diagnosis, years	1.04 (1.01–1.06)	0.003	–	–
Hb level at diagnosis, g/dl	–	–	1.32 (1.00–1.72)	0.05
Monosomal karyotype (present vs absent)	2.87 (1.36–6.05)	0.006	–	–
Complex karyotype (present vs absent)	–	–	0.41 (0.16–1.00)	0.05
*MN1/ABL1* copy numbers at diagnosis (high vs low, median cut)	2.26 (1.18–4.32)	0.01	–	–
*BAALC/ABL1* copy numbers at diagnosis (high vs low, median cut)	–	–	0.37 (0.14–0.97)	0.04

*Hb*, Hemoglobin

*HR; hazard ratio, ** OR, odds ratio, < 1 (> 1) indicate lower (higher) risk for an event for the first category listed for the dichotomous variables and for the higher values of the continuous variables

Variables considered in the models were those significant at *α* < 0.10 in univariable analyses. For EFS endpoint, variables considered were age at diagnosis, disease origin (de novo vs secondary), ELN genetic risk, normal karyotype (present vs absent), complex karyotype (present vs absent), monosomal karyotype (present vs absent), *BAALC*/*ABL1* copy numbers (high vs low, median cut) and *MN1*/*ABL1* copy numbers (high vs low, median cut). For OS endpoint, variables considered were hemoglobin level at diagnosis, ELN genetic risk, normal karyotype (present vs absent), complex karyotype (present vs absent), monosomal karyotype (present vs absent), and *BAALC*/*ABL1* copy numbers (high vs low, median cut)

**Table 3:** Multivariate analysis for patients in the outcome set receiving allogeneic HSCT (*n* = 186)

	Event free survival		Overall survival	
	HR* (95% CI)	*P*	OR** (95% CI)	*P*
Age at diagnosis	–	–	0.96 (0.93–0.98)	0.001
BM blast count at diagnosis	0.99 (0.97–1.00)	0.05	1.01 (1.00–1.02)	0.01
ELN genetic risk (adverse vs intermediate vs favorable)	1.97 (1.25–3.10)	0.004	0.71 (0.53–0.94)	0.02
Pre-HSCT *MN1/ABL1* copy numbers (high vs low, 0.30 cut)	2.99 (1.44–6.21)	0.003	–	–

*BM*, bone marrow; *ELN*, European LeukemiaNet; *HSCT*, hematopoietic stem cell transplantation

*HR, hazard ratio; **OR, odds ratio, < 1 (> 1) indicate lower (higher) risk for an event for the first category listed for the dichotomous variables and for the higher values of the continuous variables

Variables considered in the models were those significant at α < 0.10 in univariable analyses. For EFS endpoint, variables considered were age at diagnosis, disease origin (de novo vs secondary), bone marrow blast count at diagnosis, hemoglobin level at diagnosis, ELN genetic risk, complex karyotype (present vs absent), monosomal karyotype (present vs absent), *EVI1* expression status (positive vs negative), preHSCT *BAALC*/*ABL1* copy numbers (high vs low, 0.30 cut) and preHSCT *MN1*/*ABL1* copy numbers. For OS endpoint, variables considered were age at diagnosis, disease origin (de novo vs secondary), ELN genetic risk, bone marrow blast count at diagnosis, *EVI1* expression status (positive vs negative), preHSCT *BAALC*/*ABL1* copy numbers (high vs low, 0.30 cut) and preHSCT *MN1*/*ABL1* copy numbers

Similar results were observed when we restricted our analyses to patients with a normal karyotype (Supplementary Figs. S2 and S3) or patients transplanted in first CR (Supplementary Fig. S4). Additionally, we performed a landmark analysis for patients receiving chemotherapy for the first 139 days after diagnosis (median time from diagnosis to HSCT in the HSCT treated cohort) and again observed shorter EFS (*P* = 0.02) and by trend shorter OS (*P* = 0.08) for patients with high *BAALC*/*ABL1* copy numbers at diagnosis (Supplementary Fig. S5A, B) as well as shorter EFS (*P* = 0.05) and by trend shorter OS (*P* = 0.10) for patients with high *MN1*/*ABL1* copy numbers at diagnosis (Supplementary Fig. S5C, D).

Differences between patients consolidated with chemotherapy and patients receiving allogeneic HSCT are shown in the Supplementary Information and Supplementary Table S3.

## Discussion

As a result of the search for better risk stratification in AML patients with normal cytogenetics, high diagnostic expression of the AML-associated genes *BAALC* and *MN1* were shown to have independent adverse prognostic impact on CR achievement, relapse rates, EFS, and OS in younger [4, 9, 12–14, 27–29] and older [10–12] AML patients. Some later investigations also suggested a prognostic impact in AML patients with abnormal cytogenetics [30] or independently from cytogenetic groups [20, 31, 32]. Most of these studies focused on chemotherapy-based consolidation therapies or autologous HSCT with only a very small proportion of patients receiving an allogeneic—and in the majority of cases related donor—HSCT. However, there have already been some indications that the prognostic impact of diagnostic *BAALC* and *MN1* expression may be modulated by the consolidation treatment. Yoon et al. [33] analyzed a cohort of 125 cytogenetically normal AML patients of whom approximately half were consolidated with an allogeneic HSCT and did not observe a prognostic impact of high *BAALC* expression levels, which might be explained by the mixed consolidation therapies. One recent study suggested comparable EFS and OS for high and low *BAALC* expressers in the TCGA dataset for patients after allogeneic HSCT, but this analysis was limited by low patient numbers and missing data on the applied chemotherapies and conditioning regimens [20]. In a subanalysis of 48 patients receiving allogeneic HSCT, Baldus et al. [28] observed very low relapse rates irrespective of *BAALC* expression at diagnosis and suggested that high *BAALC* expressing patients might benefit from an allogeneic HSCT. With respect to diagnostic *MN1* expression, in a donor vs no donor subanalysis by Heuser et al. [4], no benefit of an allogeneic HSCT in high expressers was observed, but also this study was also restricted by low patient numbers (*n* = 39). Thus, the prognostic significance of *BAALC* and *MN1* expression levels at diagnosis in the context of an allogeneic HSCT remains to be evaluated in a large homogeneously treated and genetically well-defined patient set—which was the main objective of our study.

In contrast to previous reports that used qRT-PCR [4, 9, 13, 14, 27, 28] or microarray-based [12, 32] assays for evaluation of *BAALC* and *MN1* expression levels, we adopted a ddPCR technology. This method allows absolute quantification of gene copy numbers at high sensitivity, specificity, and reproducibility without the need of standard curves [21] and enabled us to establish an assay sufficient for a routine clinical assessment of *BAALC* and *MN1* expression. In a subset of 110 patients, we observed a high correlation between qRT-PCR and ddPCR results for both gene expressions (Fig. 1) underlining the feasibility of our ddPCR assays.

The observed associations of diagnostic *BAALC* and *MN1* copy numbers with clinical and genetic parameter stand in line with previously published analyses [4, 9–14, 20, 27, 31, 32]. As previously reported [13], high *BAALC* and *MN1* expression correlated with each other, as well as with a high expression of immature markers such as CD34 [4, 9, 10, 31] and CD117 [9]. Additionally, we observed an association of high *BAALC*/*ABL1* and *MN1*/*ABL1* copy numbers with the CD34+/CD38− cell burden, and *GPR56*, which match the suggestions by Liu et al. [34] that *MN1* overexpression might contribute to an expansion of the leukemic stem cell population. High *BAALC*/*ABL1* and *MN1*/*ABL1* copy numbers correlated with a specific immunophenotype, including a lower expression of mature myeloid antigens, e.g., CD11b or CD15, which have already been described for *BAALC* [27], and higher expression of antigens associated with T cell differentiation. Additionally, both high *BAALC*/*ABL1* and *MN1*/*ABL1* expressing patients showed lower CD33 expression, which might have clinical consequences when considering CD33-targeted treatment approaches [35]. We also observed the previously reported association of high *BAALC* and *MN1* levels with lower white blood counts [9, 11, 14], immature FAB types [12, 14], abnormal cytogenetics [20, 32], *NPM1* wild-type [9–13], as well as mutated *CEBPA* for high *MN1* expressers [12]. Within the TCGA data set an association of high *BAALC* expression levels with mutated *RUNX1* was described [20] that we observed for both high *BAALC* and high *MN1* expressing patients. While we did not find an association of high *BAALC* levels with wild-type *PTPN11* [20], there was a not yet reported lower incidence of *DNMT3A* mutations for high *BAALC* expressers, as well as a trend for less *TET2* mutations in both high *BAALC* and *MN1* expressing patients.

As expected, high *BAALC* and *MN1* copy numbers associated with inferior outcomes in AML patients after chemotherapy-based consolidation. In contrast, within the large group of patients consolidated with an allogeneic HSCT, we observed no prognostic impact of *BAALC* or *MN1* copy numbers at diagnosis, which was also seen in separate analyses for patients with a normal karyotype and patients transplanted in first CR. Noteworthy, also the cumulative incidences of relapse and nonrelapse mortality according to *BAALC*/*ABL1* and *MN1*/*ABL1* copy numbers did not differ after allogeneic HSCT (Supplementary Fig. S6).

This is especially interesting because even though for some prognostic markers allogeneic HSCT has been described to improve outcomes, the prognostic impact of most of these markers retain their prognostic impact in the HSCT context [23, 36, 37]. However, patients with high *BAALC* or *MN1* expression at diagnosis—both markers repeatedly published to confer inferior prognosis in chemotherapy-consolidated AML patients—might benefit from an allogeneic HSCT as consolidation therapy. Noteworthy, genes involved in antigen processing and expression—among those genes encoding for MHC class I and MHC class II molecules—correlate positively with *MN1* gene expression signatures [13]*.* This associated gene expression might support immunologic GvL effects after HSCT to contribute to better outcomes in AML patients with high *MN1* expression.

We previously described the prognostic utility of *BAALC*/*ABL1* and *MN1*/*ABL1* copy numbers for risk stratification in remission prior to an allogeneic HSCT—which are likely to reflect residual disease burden at this time point [15, 16]. In the here-presented patient set, we also observed a strong impact on EFS and OS after HSCT according to preHSCT *BAALC*/*ABL1* (Supplementary Fig. S7A, B) and *MN1*/*ABL1* copy numbers (Supplementary Fig. S8A, B). Noteworthy, there was no correlation between *BAALC*/*ABL1* and *MN1/ABL1* copy numbers at diagnosis and in peripheral blood remission samples prior to HSCT (Supplementary Fig. S9). The prognostic impact of preHSCT *BAALC*/*ABL1* and *MN1*/*ABL1* copy numbers was independent of the diagnostic *BAALC*/*ABL1* (Supplementary Fig. S7C–F) or *MN1*/*ABL1* copy numbers (Supplementary Fig. S8C–F). PreHSCT *BAALC*/*ABL1* and *MN1*/*ABL1* copy numbers may have the highest prognostic value in patients with low copy numbers at diagnosis as this may result in higher assay sensitivity (indicated in Supplementary Figs. S7C–F and S8C–F), but larger analyses are needed to confirm this assumption. In contrast, also in patients with high or low preHSCT *BAALC*/*ABL1* or *MN1*/*ABL1* copy numbers, diagnostic *BAALC*/*ABL1* or *MN1*/*ABL1* copy numbers did not impact outcome (Supplementary Fig. S10).

Taken together, these data indicate that in the context of an allogeneic HSCT, the diagnostic *BAALC* or *MN1* expression levels do not impact prognosis. However, independent of the diagnostic *BAALC* or *MN1* expression levels, the assessment of both gene copy numbers in remission prior to allogeneic HSCT allow for relevant risk stratification. This further confirms previous data showing that outcomes of AML patients undergoing allogeneic HSCT remain the most favorable if patients are measurable residual disease negative prior to start of conditioning regimens [15, 16, 38–41].

In conclusion, we show that the adverse prognostic impact of high *BAALC* and *MN1* expression levels at diagnosis is mitigated in AML patients undergoing allogeneic HSCT. In contrast, in patients receiving chemotherapy alone, we could confirm the described inferior outcomes for individuals with high *BAALC* or *MN1* expression at diagnosis. Our data indicate that patients with high *BAALC* or *MN1* expression at diagnosis might benefit from an allogeneic HSCT which would help to individualize treatment of these patients. Prospective analyses would be helpful to further confirm this observation.

## Electronic supplementary material

ESM 1 (DOCX 3399 kb)
