# Supplementary material for: Allogeneic stem cell transplantation mitigates the adverse prognostic impact of high diagnostic BAALC and MN1 expression in AML
Source: Ann Hematol. 2020 Aug 29;99(10):2417–27. doi: 10.1007/s00277-020-04235-8 (PMC7481166; doi:10.1007/s00277-020-04235-8)
Supplement: Supplementary file 1 — (DOCX 3399 kb) [file 277_2020_4235_MOESM1_ESM.docx]

**SUPPLEMENTARY INFORMATION**

**to Jentzsch *et al.*: „Allogeneic stem cell transplantation mitigates the adverse prognostic impact of high diagnostic *BAALC* and *MN1* expression in AML”**

**SUPPLEMENTARY METHODS**

**Further Patients Characteristics**

Additional clinical, genetic, molecular, and flow cytometry information at diagnosis for the whole patient cohort are shown in Supplementary Table S1. Hematopoietic stem cell transplantation (HSCT) related information for transplanted patients in the outcome set are shown in Supplementary Table S2.

**Induction therapy protocols of patients in the outcome set**

In the subgroup of acute myeloid leukemia (AML) patients younger than 60 years at diagnosis (n=116), 109 patients received chemotherapy according to the AML 2002 study (OSHO #061) [1], five patients received chemotherapy within the Ratify trial [2], one patient received chemotherapy within the Unify trial (ClinicalTrials.gov Identifier: NCT03512197) and one patient was diagnosed with AML as a child and treated within the AML BFM-2014 study [3]. Among AML patients older than 60 years at diagnosis (n=147), 136 patients were treated within the AML 2004 study (OSHO #069) [4], and eleven patients were treated within the OSHO #083 protocol. All patients in the outcome set received at least one cycle of intensive chemotherapy.

**Allogeneic HSCT of patients in the outcome set**

As this is a retrospective analysis of patients diagnosed between 2000 and 2018, reasons for the applied consolidation treatment were heterogeneous and changed over time according to the current treatment recommendations for AML patients (e.g. according to risk stratification: MRC, ELN 2010, ELN 2017) [5-7] as well as according to study protocols (HCT Versus CT in Elderly AML, ClinicalTrials.gov Identifier: NCT00766779). In general, patients outside of clinical HSCT trials with adequate performance status (ECOG ≤ 2) and intermediate or adverse prognosis according to the respective risk stratification were recommended to undergo allogeneic HSCT in first complete remission (CR) if a suitable donor was available. In second CR, all patients with adequate performance status (ECOG ≤ 2) were recommended to undergo allogeneic HSCT. In patients receiving HSCT, reasons for non-myeloablative (NMA) HSCT as opposed to myeloablative conditioning (MAC) were age over 50 years if receiving unrelated HSCT and over 55 years if receiving related HSCT, prior autologous HSCT (n=3) or active infections (n=2).

**Prevention of graft-versus-host disease**

Prevention of graft-versus-host disease (GvHD) was different according to the two conditioning regimes used. All patients receiving MAC were treated with cyclosporine A (CyA), starting intravenously with 5 mg/kg body weight (BW) in two daily doses from day -1. Blood levels of CyA were measured from day 0 and doses were adjusted for target levels of 200 ng/ml. Patients also received methotrexate 15 mg intravenously on days +1, +3, +6 and +11 after HSCT. Furthermore, patients with an unrelated donor additionally received *in vivo* T-cell depletion with thymoglobulin 2 mg/kg BW per day on days -3 to -1.

All patients with NMA-HSCT received a starting dose of 5 mg/kg BW CyA in two daily doses from day -1. Blood levels of CyA were measured from day 0 and doses were adjusted for target levels of 200 ng/ml. Additionally, patients with NMA conditioning received mycophenolate mofetil (MMF) 3 g per day in three daily doses if receiving unrelated HSCT or 2 g per day in two daily doses if receiving related HSCT. CyA was reduced starting on day +84 or day +180 following related or unrelated HSCT, respectively, and MMF was stopped at day +28 following related HSCT and tapered from days +40 to +96 following unrelated HSCT [8].

For all patients after MAC- or NMA-HSCT, immunosuppression was prolonged or extended with systemic steroids in cases of GvHD (grade > 2 according to Glucksberg grading system [9]) or rapidly reduced in patients who relapsed (≥ 5% blasts in bone marrow). Patients were evaluated for incidence of acute GvHD (aGvHD) and chronic GvHD (cGvHD), using established criteria of the Glucksberg grading system [9]. Requirement for aGvHD was engraftment while requirement for cGvHD was engraftment and survival for at least 100 days after HSCT. As this is a retrospective analysis, we lack GvHD grading according to the NIH consensus criteria [10].

**Definition of complete remission**

CR was defined as the presence of <5% of blasts in bone marrow (BM), neutrophils >1.0 x 10^9^/L, platelets >100 x 10^9^/L, absence of blasts with Auer rods, independence of blood transfusion and no extramedullary disease [6]. CR with incomplete peripheral recovery (CRi) was defined as CR with platelets <100 x 10^9^/L or neutrophils <1.0 x 10^9^/L. In patients receiving allogeneic HSCT, the presence of CR or CRi was confirmed within 28 days prior to HSCT by bone marrow and peripheral blood analysis.

**qRT-PCR Analysis of gene expression levels**

Mononuclear cells were isolated from pre-treatment bone marrow for all patients. Total RNA was extracted from 1x10^7^ cells and processed to complementary DNA as previously described [11]. qRT-PCR of *GPR56* (Hs00173754_m1), *BAALC* (Hs00227249_m1), *MN1* (Hs00159202_m1), *EVI1* (Hs00602795_m1), *ABL1* (Hs01104728_m1) and *18S* (Hs99999901_s1) was performed with Taqman gene expression assays (Applied Biosystems, Carlsbad, CA) following the manufacturer’s protocols. *GPR56*, *BAALC,* and *MN1* expression was normalized to *ABL1* as internal control. The comparative cycle threshold (ΔΔC_T_) method was used for relative quantification of gene expression as previously described [12]. The to *18S* normalized expression of *EVI1* in the cell line SKOV3 was used to define *EVI1* positive expressers, i.e. patients with an expression higher than 0.1 relative to the *EVI1* expression of SKOV3 were labeled *EVI1* positive [13].

**Multivariate analyses**

We constructed two multivariable proportional hazard models for event free survival (EFS) and overall survival (OS) to evaluate the impact of *BAALC/ABL1* and *MN1/ABL1* copy numbers at diagnosis in patients treated with chemotherapy or allogeneic HSCT by adjusting for other variables.

In addition to *BAALC/ABL1* and *MN1/ABL1* copy numbers at diagnosis (low *vs* high, median cut), the following variables were considered for multivariable analyses: sex, hemoglobin count, platelet count, white blood cell count, blast percentages in peripheral blood and bone marrow at diagnosis, disease origin (*de novo* vs secondary), European LeukemiaNet (ELN) 2017 genetic risk, presence of a complex karyotype, presence of a monosomal karyotype, mutation status of the tyrosine kinase domain of the *FLT3* gene (*FLT3*-TKD), *EVI1* expression status (positive *vs* negative), age at HSCT, pre-HSCT *BAALC/ABL1* copy numbers, pre-HSCT *MN1/ABL1* copy numbers, disease status at HSCT (CR *vs* CRi), cytomegalovirus (CMV) status of recipient and donor (high risk [+/-] *vs* all others), HLA match (antigen match *vs* mismatch), HLA donor type (related *vs* unrelated) and sex of the donor. Of these, variables significant at α=.10 in univariable analyses were considered for multivariable analyses. Variables were obtained using forward selection analysis and models were compared using the Akaike information criterion (AIC) to identify the statistically preferred model. For both endpoints, hazard/odds ratios with their corresponding 95% confidence intervals are indicated for every significant prognostic factor of the final model.

**SUPPLEMENTARY RESULTS**

**Patients in the outcome set treated with chemotherapy alone or consolidated with allogeneic HSCT**

Compared to patients receiving an allogeneic HSCT, patients in the outcome set who were treated with chemotherapy alone were older (*P*<.001) and more likely to harbor a complex karyotype (*P*=.02). All other analyzed parameter, including ELN genetic risk (*P*=.13), disease origin (*P*=.56) and diagnostic *BAALC/ABL1* (*P*=.89) and *MN1/ABL1* copy numbers (*P*=.77) were not significantly different between patients receiving chemotherapy or allogeneic HSCT (Supplementary Table S3).

**Subgroup analyses for the prognostic value of *BAALC/ABL1* and *MN1/ABL1* copy at diagnosis**

To gain further insight into the prognostic significance of *BAALC/ABL1* and *MN1/ABL1* copy numbers at diagnosis we performed subgroup analyses for patients with a normal karyotype, or patients transplanted in CR1.

Despite restricted patient numbers, we observed a separation of the EFS and OS curves for the 33 patients with normal karyotype treated with chemotherapy alone (Supplementary Figure S2A,B and S3A,B). In contrast, for patients with normal karyotype consolidated with an allogeneic HSCT, there was no different EFS (*P*=.30 and *P*=.60, Supplementary Figure 2C and 3C) or OS (*P*=.90 and *P*=.40, Supplementary Figure 2D and 3D) according to *BAALC/ABL1* or *MN1/ABL1* copy numbers at diagnosis, respectively. Similarly, when we restricted our analysis to patients receiving HSCT in first CR, we again observed no significant impact on EFS (*P*=.30 and *P*=.40, Supplementary Figure 4A,C) or OS (*P*=.30 and *P*=.30, Supplementary Figure 4B,D) according to *BAALC/ABL1* or *MN1/ABL1* copy numbers at diagnosis, respectively.

**Prognostic impact of *BAALC/ABL1* and *MN1/ABL1* copy numbers prior to allogeneic HSCT**

We previously showed the prognostic significance of pre-HSCT *BAALC/ABL1* [14] and *MN1/ABL1* copy numbers [15] on outcome after allogeneic HSCT. For pre-HSCT *BAALC/ABL1* copy numbers our data indicated that this prognostic impact was independent of the copy numbers at diagnosis but this analysis was restricted by low patient numbers (16 patients with low and 35 with high diagnostic *BAALC/ABL1* copy numbers) [14].

In the here presented patient set, of the patients with pre-HSCT data available, 28 had high and 44 had low pre-HSCT *BAALC/ABL1* copy numbers while 21 had high and 55 had low pre-HSCT *MN1/ABL1* copy numbers. Using the previously published cut-offs [14,15], high pre-HSCT *BAALC/ABL1* copy numbers and high pre-HSCT *MN1/ABL1* copy numbers again associated with shorter EFS (*P*<.001 and *P*<.001, respectively) and shorter OS (*P*<.001 and *P*=.002, respectively, Supplementary Figure S7A,B and S8A,B).

In patients with low *BAALC/ABL1* copy numbers at diagnosis, high pre-HSCT *BAALC* associated with shorter EFS (*P*=.005, Supplementary Figure S7C) and shorter OS (*P*<.001, Supplementary Figure S7D). Similarly, in patients with high *BAALC/ABL1* copy numbers at diagnosis we observed a shorter EFS (*P*=.05, Supplementary Figure S7E) and a trend for shorter OS (*P*=.06, Supplementary Figure S7F) for patients with high pre-HSCT *BAALC/ABL1* copy numbers. In patients with low *MN1/ABL1* copy numbers at diagnosis, high pre-HSCT *MN1/ABL1* copy numbers associated with shorter EFS (*P*=.04, Supplementary Figure S8C) and shorter OS (*P*=.005, Supplementary Figure S8D). In patients with high *MN1/ABL1* copy numbers at diagnosis, high *MN1/ABL1* copy numbers associated with shorter EFS (*P*=.01, Supplementary Figure S8E) while despite a separation of the curves, OS was not significantly different (*P*=.30, Supplementary Figure S8F). In conclusion, this extended data further underlines the utility of pre-HSCT *BAALC/ABL1* and *MN1/ABL1* copy numbers for residual disease detection and their independence from copy numbers at diagnosis.

**Correlation between *BAALC/ABL1* and *MN1/ABL1* copy numbers at diagnosis and prior to HSCT**

Consecutive *BAALC/ABL1* and *MN1/ABL1* copy numbers at diagnosis (assessed in bone marrow) and prior to HSCT (assessed in peripheral blood) were available in 77 and 76 patients, respectively. We observed no correlation between pre-treatment and pre-HSCT *BAALC/ABL1* copy numbers (Pearson correlation coefficient *r*=-.07, Supplementary Figure S9A) or *MN1/ABL1* copy numbers (Pearson correlation coefficient *r*=.03, Supplementary Figure S9B).

**SUPPLEMENTARY REFERENCES**

**1** Büchner T, Schlenk RF, Schaich M, Döhner K, Krahl R, Krauter J *et al*. Acute Myeloid Leukemia (AML): different treatment strategies versus a common standard arm-combined prospective analysis by the German AML Intergroup. *J Clin Oncol* 2012; **30**: 3604-3610.

**2** Stone RM, Mandrekar SJ, Sanford BL, Laumann K, Geyer S, Bloomfield CD *et al*. Midostaurin plus Chemotherapy for Acute Myeloid Leukemia with a *FLT3* Mutation. *N Engl J Med* 2017; **377**: 454-464.

**3** Creutzig U, Zimmermann M, Bourquin JP, Dworzak MN, Fleischhack G, Graf N *et al*. Randomized trial comparing liposomal daunorubicin with idarubicin as induction for pediatric acute myeloid leukemia: results from Study AML-BFM 2004. *Blood* 2013; **122**: 37-43.

**4** Niederwieser D, Hoffmann VS, Pfirrmann M, Al-Ali HK, Schwind S, Vucinic V *et al.* Comparison of Treatment Strategies in Patients over 60 Years with AML: Final Analysis of a Prospective Randomized German AML Intergroup Study. [abstract]. *Blood* 2016; 128:1066. Abstract 1066.

**5** Grimwade D, Walker H, Oliver F, Wheatley K, Harrison C, Harrison G *et al*. The importance of diagnostic cytogenetics on outcome in AML: analysis of 1,612 patients entered into the MRC AML 10 trial. The Medical Research Council Adult and Children's Leukaemia Working Parties. *Blood* 1998; **92**: 2322-2333.

**6** Döhner H, Estey EH, Amadori S, Appelbaum FR, Büchner T, Burnett AK *et al*. Diagnosis and management of acute myeloid leukemia in adults: Recommendations from an international expert panel, on behalf of the European Leukemia-Net. *Blood* 2010; **115**: 453-474.

**7** Döhner H, Estey E, Grimwade D, Amadori S, Appelbaum FR, Büchner T *et al*. Diagnosis and management of AML in adults: 2017 ELN recommendations from an international expert panel. *Blood* 2017; **129**: 424-447.

**8** Niederwieser D, Maris M, Shizuru JA, Petersdorf E, Hegenbart U, Sandmaier BM *et al*. Low-dose total body irradiation (TBI) and fludarabine followed by hematopoietic cell transplantation (HCT) from HLA-matched or mismatched unrelated donors and postgrafting immunosuppression with cyclosporine and mycophenolate mofetil (MMF) can induce durable complete chimerism and sustained remissions in patients with hematological diseases. *Blood* 2003; **101**: 1620-1629.

**9** Glucksberg H, Storb R, Fefer A, Buckner CD, Neiman PE, Clift RA *et al*. Clinical manifestations of graft-versus-host disease in human recipients of marrow from HLA matched sibling donors. *Transplantation* 1974; **18**: 295–304.

**10** Vigorito AC, Campregher PV, Storer BE, Carpenter PA, Moravec CK, Kiem HP *et al*; National Institutes of Health. Evaluation of NIH consensus criteria for classification of late acute and chronic GVHD. *Blood* 2009; **114**: 702-8.

**11** Lange T, Niederwieser DW, Deininger MW. Residual disease in chronic myeloid leukemia after induction of molecular remission. *N Engl J Med* 2003; **349**: 1483–1484.

**12** Mims A, Walker AR, Huang X, Sun J, Wang H, Santhanam R *et al*. Increased anti-leukemic activity of decitabine via AR-42-induced upregulation of miR-29b: a novel epigenetic-targeting approach in acute myeloid leukemia. *Leukemia* 2013; **27**: 871-878.

**13** Gröschel S, Lugthart S, Schlenk RF, Valk PJ, Eiwen K, Goudswaard C *et al*. High *EVI1* Expression Predicts Outcome in Younger Adult Patients With Acute Myeloid Leukemia and Is Associated With Distinct Cytogenetic Abnormalities. *J Clin Oncol* 2010; **28**: 2101-2107.

**14** Jentzsch M, Bill M, Grimm J, Schulz J, Goldmann K, Beinicke S *et al*. High *BAALC* copy numbers in peripheral blood prior to allogeneic transplantation predict early relapse in acute myeloid leukemia patients. *Oncotarget* 2017; **8**: 87944-87954.

**15** Jentzsch M, Bill M, Grimm J, Schulz J, Beinicke S, Häntschel J *et al*. Prognostic Impact of Blood *MN1* Copy Numbers Before Allogeneic Stem Cell Transplantation in Patients With Acute Myeloid Leukemia. *HemaSphere* 2019; **3**: e167.

**Supplemental Tables**

**Table S1:** **Additional clinic, genetic and flow cytometry characteristics at diagnosis for all patients according to *BAALC* (high *vs* low, median cut) and *MN1* (high *vs* low, median cut) *copy numbers* at diagnosis (n=302).**

|  | **All patients**  **n=302** | **low *BAALC* copy numbers**  **n=151** | **high *BAALC* copy numbers**  **n=151** | ***P*** | **low *MN1* copy numbers**  **n=51** | **high *MN1* copy numbers**  **n=51** | ***P*** |
| --- | --- | --- | --- | --- | --- | --- | --- |
| **Clinical information at diagnosis** | | | | | | | |
| FAB type, n (%)  M0  M1  M2  M4  M4eo  M5  M6  M7 | 6  26  140  30  17  35  9  10 | 2 (1)  4 (3)  72 (52)  17 (12)  4 (3)  28 (20)  6 (4)  6 (4) | 4 (3)  22 (16)  68 (51)  13 (10)  13 (10)  7 (5)  3 (2)  4 (3) | .44  <.001  1  .57  .02  <.001  .50  .75 | 1 (1)  6 (4)  74 (54)  13 (10)  4 (3)  26 (19)  7 (5)  5 (4) | 5 (4)  20 (15)  66 (48)  17 (12)  13 (9)  9 (7)  2 (15)  5 (4) | .12  .004  .40  .45  .03  .003  .17  1 |
| **Genetic information at diagnosis** | | | | | | | |
| CBF-AML, n (%)  absent  present | 263  25 | 142 (100)  0 (0) | 120 (83)  25 (27) | <.001 | 138 (97)  4 (3) | 124 (86)  21 (14) | .001 |
| Trisomy 8, n (%)  absent  present | 260  36 | 126 (85)  22 (15) | 134 (91)  14 (9) | .21 | 131 (89)  17 (11) | 129 (87)  17 (13) | .86 |
| del(5)/del(5q), n (%)  absent  present | 269  27 | 143 (97)  5 (3) | 126 (85)  22 (15) | .001 | 141 (95)  7 (5) | 128 (86)  20 (14) | .01 |
| del(7)/del(7q), n (%)  absent  present | 244  41 | 137 (93)  10 (7) | 117 (79)  31 (21) | .001 | 137 (93)  10 (7) | 117 (79)  31 (21) | .001 |
| Monosomal karyotype, n (%)  absent  present | 246  41 | 129 (91)  13 (9) | 117 (81)  28 (19) | .02 | 127 (89)  15 (11) | 119 (82)  26 (18) | .09 |
| Complex karyotype, n (%)  absent  present | 235  52 | 124 (87)  18 (13) | 111 (77)  34 (23) | .02 | 121 (85)  21 (15) | 114 (79)  31 (21) | .17 |
| *FLT3*-TKD, n (%)  wild-type  mutated | 257  33 | 131 (92)  12 (8) | 126 (86)  21 (14) | .14 | 130 (90)  14 (10) | 127 (87)  19 (13) | .46 |
| *IDH1*, n (%)  wild-type  mutated | 136  16 | 65 (86)  11 (14) | 71 (93)  5 (7) | .19 | 61 (86)  10 (14) | 75 (93)  6 (7) | .20 |
| *IDH2*, n (%)  wild-type  mutated | 128  24 | 61 (80)  15 (20) | 67 (88)  9 (12) | .27 | 58 (82)  13 (18) | 70 (86)  11 (14) | .51 |
| *DNMT3A*, n (%)  wild-type  mutated | 107  20 | 46 (77)  14 (23) | 61 (91)  6 (9) | .03 | 50 (79)  13 (21) | 57 (89)  7 (11) | .15 |
| *TET2*, n (%)  wild-type  mutated | 72  17 | 33 (73)  12 (27) | 39 (89)  5 (11) | .10 | 33 (73)  12 (27) | 39 (89)  5 (11) | .10 |
| *EVI1* expression status, n (%)  negative  positive | 125  32 | 66 (86)  11 (14) | 59 (74)  21 (26) | .08 | 65 (84)  12 (16) | 60 (75)  20 (25) | .17 |
| *GPR56* expression  median  range | 0.09  0.0009-55.82 | 0.04  0.001-12.96 | 0.12  0.0001-55.82 | <.001 | 0.05  0.0001-12.96 | 0.12  0.0002-55.8 | <.001 |
| **Surface antigen expression at diagnosis** | | | | | | | |
| BM CD117 expression, %  median  range | 34  0.3-96 | 24  0.3-95 | 39  0.5-96 | <.001 | 26  0.3-95 | 37  0.5-96 | <.001 |
| BM CD38 expression, %  median  range | 75  0.5-98 | 78  0.5-98 | 75  4-98 | .57 | 75  0.5-98 | 76  4-98 | .87 |
| BM CD45 expression, %  median  range | 93  6-100 | 93  6-100 | 93  14-99 | 1 | 93  6-100 | 93  32-99 | .87 |
| BM CD11b expression, %  median  range | 19  0.5-95 | 24  0.5-95 | 16  0.5-92 | .01 | 22  0.5-95 | 19  0.5-92 | .24 |
| BM CD13 expression, %  median  range | 63  2-96 | 60  2-96 | 66  2-96 | .04 | 58  2-96 | 66  7-96 | .007 |
| BM CD15 expression, %  median  range | 31  1-96 | 36  1-96 | 30  1-89 | .07 | 36  1-96 | 27  1-89 | .45 |
| BM CD33 expression, %  median  range | 69  2-98 | 77  2-98 | 62  6-98 | .001 | 75  8-98 | 55  2-94 | .05 |
| BM CD65 expression, %  median  range | 18  0.5-91 | 18  0.5-91 | 18  0.5-90 | .31 | 18  0.5-91 | 18  0.5-90 | .32 |
| BM CD14 expression, %  median  range | 3  0.5-91 | 3  0.5-91 | 3  0.5-86 | .61 | 2  0.5-91 | 3  0.5-86 | .72 |
| BM CD64 expression, %  median  range | 17  0-98 | 34  0.5-98 | 10  0-95 | <.001 | 30  0.5-98 | 11  0-95 | .001 |
| BM CD61 expression, %  median  range | 4  0-67 | 3  0.5-66 | 5  0-67 | .58 | 3  0.5-66 | 5  0.5-67 | .20 |
| BM Glykophorin A expression, %  median  range | 8  0-90 | 8  0-90 | 9  0.5-62 | .89 | 7  0-90 | 10  0.5-62 | .68 |
| BM CD2 expression, %  median  range | 12  1-97 | 10  1-97 | 17  2-92 | <.001 | 9  1-97 | 17  2-75 | <.001 |
| BM CD7 expression, %  median  range | 14  1-96 | 10  1-95 | 19  2-96 | <.001 | 9  1-95 | 20  2-96 | <.001 |
| BM CD56 expression, %  median  range | 9  0-98 | 8  0.5-96 | 10  0-98 | .99 | 10  0-96 | 9  0.5-98 | .46 |
| **Treatment** | | | | | | | |
| Consolidation therapy (outcome set), n (%)  chemotherapy  allogeneic HSCT | 77  186 | 40 (31)  90 (48) | 37 (28)  96 (72) | .60 | 40 (31)  90 (69) | 37 (28)  96 (72) | .60 |

*Abbreviations: BAALC, brain and acute leukemia cytogenetic gene; BM, bone marrow; DNMT3A, DNA-methyltransferase 3A gene; EVI1, ecotropic virus integration-1 gene; FAB, french american british; FLT3-TKD, tyrosine kinase domain of the FLT3 gene; GPR56, G protein-coupled receptor 56; HLA, human leukocyte antigen; HSCT, hematopoietic stem cell transplantation; IDH1, isocitrat dehydrogenase 1 gene; IDH2, isocitrat dehydrogenase 2 gene; MN1, meningioma 1 gene; MRD, measurable residual disease; NPM1, nucleophosmin 1 gene; TET2, Ten-Eleven Translocation-2.*

**Table S2:** **HSCT-associated characteristics for patients in the outcome set receiving allogeneic HSCT for consolidation therapy according to *BAALC/ABL1* (high *vs* low, median cut) and *MN1/ABL1* (high *vs* low, median cut) *copy numbers* at diagnosis (n=186).**

|  | **All patients**  **n=186** | **low *BAALC* copy numbers**  **n=90** | **high *BAALC* copy numbers**  **n=96** | ***P*** | **low *MN1* copy numbers**  **n=90** | **high *MN1* copy numbers**  **n=96** | ***P*** |
| --- | --- | --- | --- | --- | --- | --- | --- |
| **HSCT-related characteristics** | | | | | | | |
| Remission at HSCT, n (%)  CR1  CR2  CRi | 131  31  24 | 62 (69)  19 (21)  9 (10) | 69 (72)  12 (13)  15 (16) | .51 | 61 (68)  18 (20)  11 (12) | 70 (73)  13 (14)  13 (14) | .51 |
| Pre-HSCT *BAALC/ABL1* copy numbers (MRD), n (%)  low  high | 49  28 | 23 (68)  11 (32) | 26 (60)  17 (40) | .64 | 22 (67)  11 (33) | 27 (61)  17 (39) | .81 |
| Pre-HSCT *MN1/ABL1* copy numbers (MRD), n (%)  low  high | 55  21 | 22 (65)  12 (35) | 33 (79)  9 (21) | .21 | 21 (64)  12 (36) | 34 (79)  9 (21) | .20 |
| donor type, n (%)  HLA matched related  HLA matched unrelated  HLA mismatched unrelated | 42  108  36 | 23 (26)  52 (58)  15 (17) | 19 (20)  56 (58)  21 (22) | .53 | 23 (26)  52 (58)  15 (17) | 19 (20)  56 (58)  21 (22) | .53 |
| donor & recipient sex, n (%)  no female into male  female into male | 116  21 | 74 (83)  15 (17) | 82 (87)  12 (13) | .53 | 74 (84)  14 (16) | 82 (86)  13 (14) | .68 |
| CMV status, n (%)  recipient + / donor –  all others | 76  107 | 29 (33)  59 (67) | 47 (49)  48 (51) | .02 | 26 (30)  61 (70) | 50 (52)  46 (48) | .002 |
| aGvHD ≥ grade 2, n (%)  absent  present | 116  43 | 59 (75)  20 (25) | 57 (71)  23 (29) | .72 | 58 (73)  21 (27) | 58 (73)  22 (28) | 1 |
| cGvHD, n (%)  absent  limited  extended | 45  21  71 | 19 (29)  13 (20)  34 (52) | 26 (37)  8 (11)  37 (52) | .32 | 21 (33)  11 (17)  32 (50) | 24 (33)  10 (14)  39 (53) | .87 |

*Abbreviations: aGvHD, acute graft versus host disease; BAALC, brain and acute leukemia cytogenetic gene; cGvHD, chronic graft versus host disease; CMV, cytomegalovirus; CR, complete remission; CRi, CR with incomplete peripheral recovery; HLA, human leukocyte antigen; HSCT, hematopoietic stem cell transplantation; MN1, meningioma 1 gene; MRD, measurable residual disease.*

**Table S3: Comparison of patients in the outcome set receiving chemotherapy *vs* allogeneic HSCT (n=263)**

|  | **All patients**  **n=263** | **Chemotherapy cohort**  **n=77** | **HSCT cohort**  **n=186** | ***P*** |
| --- | --- | --- | --- | --- |
| **Clinical information at diagnosis** | | | | |
| Age at diagnosis, years  median  range | 61.5  14.5-82.7 | 67.0  19.7-82.7 | 60.3  14.5-75.8 | .59 |
| Sex, n (%)  male  female | 139  134 | 40 (52)  37 (48) | 89 (48)  97 (52) | .59 |
| Disease origin, n (%)  secondary  *de novo* | 79  183 | 25 (33)  51 (67) | 54 (29)  132 (71) | .56 |
| Hemoglobin, g/dL  median  range | 8.8  4.3-14.9 | 8.7  4.9-11.8 | 8.9  4.3-14.9 | .40 |
| Platelet count, x 10^9^/L  median  range | 58  2-391 | 54  2-391 | 63  2-327 | .61 |
| WBC, x 10^9^/L  median  range | 9.2  0.6-385 | 14.7  0.6-304 | 7.5  0.7-385 | .19 |
| Blood blasts, %  median  range | 26  0-98 | 25  0-96 | 27  0-98 | .62 |
| BM blasts, %  median  range | 54  0-95 | 50  16-95 | 55  0-95 | .15 |
| **Genetic information at diagnosis** | | | | |
| Normal karyotype, n (%)  absent  present | 137  113 | 40 (55)  33 (45) | 97 (55)  80 (45) | 1 |
| Complex karyotype, n (%)  absent  present | 207  41 | 55 (74)  19 (26) | 152 (87)  22 (13) | .02 |
| Monosomal karyotype, n (%)  absent  present | 214  34 | 60 (81)  14 (19) | 154 (89)  20 (11) | .16 |
| ELN2017 group, n (%)  favorable  intermediate  adverse | 86  60  88 | 25 (44)  9 (16)  23 (40) | 61 (34)  51 (29)  65 (37) | .13 |
| *NPM1*, n (%)  wild-type  mutated | 185  74 | 50 (68)  23 (32) | 135 (73)  51 (27) | .54 |
| *CEBPA*, n (%)  wild-type  mutated | 179  35 | 26 (96)  1 (4) | 153 (86)  24 (14) | .21 |
| *FLT3*-ITD, n (%)  absent  present | 212  47 | 59 (81)  14 (19) | 153 (82)  33 (18) | .86 |
| *BAALC/ABL1* copy numbers  median  range | 0.26  0-70.16 | 0.23  0.007-6.75 | 0.26  0-70.16 | .89 |
| *MN1/ABL1* copy numbers  median  range | 0.25  0.003-75.15 | 0.23  0.02-7.81 | 0.27  0.003-75.15 | .77 |

*Abbreviations: BM, bone marrow; BAALC, brain and acute leukemia cytogenetic gene; CEBPA, CCAAT/enhancer-binding protein alpha gene; DNMT3A, DNA (cytosine-5)-methyltransferase 3A gene; ELN, European Leukemia Net; FLT3-ITD, internal tandem duplication of the FLT3 gene; Hb, hemoglobin; MN1, meningioma 1 gene; NPM1, nucleophosmin 1 gene; PB, peripheral blood; WBC, white blood count.*

**Supplementary Figures**

**Supplementary Figure S1**

**
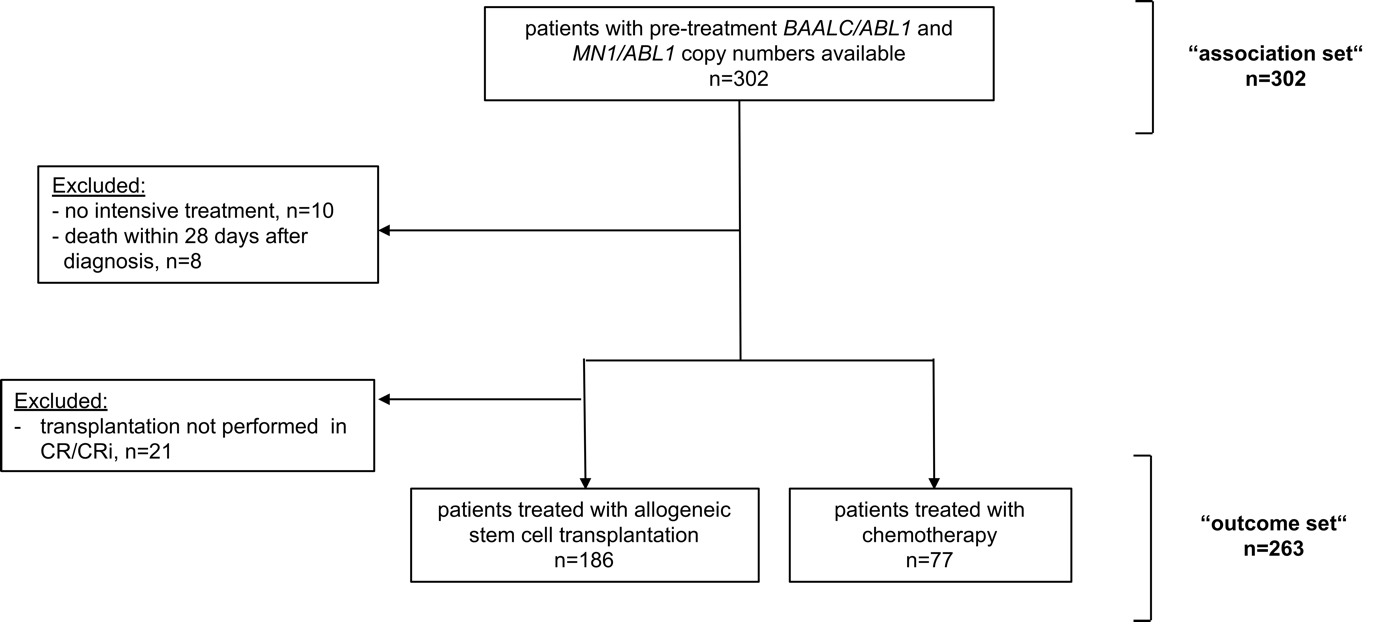
**

**Supplementary Figure 1:** Overview of AML patients included in the “association set” and “outcome set” of this study.

**Supplementary Figure S2**

**Supplementary Figure S2: Outcome according to *BAALC/ABL1* at diagnosis in AML patients with a normal karyotype (“outcome set”, n=113). (A)** Event Free Survival and **(B)** Overall Survival according in patients receiving chemotherapy alone and **(C)** Event Free Survival and **(D)** Overall Survival in patients consolidated with an allogeneic stem cell transplantation in CR/CRi.

**Supplementary Figure S3**

**Supplementary Figure S3: Outcome according to *MN1/ABL1* at diagnosis in AML patients with a normal karyotype (“outcome set”, n=113). (A)** Event Free Survival and **(B)** Overall Survival according in patients receiving chemotherapy alone and **(C)** Event Free Survival and **(D)** Overall Survival in patients consolidated with an allogeneic stem cell transplantation in CR/CRi.

**Supplementary Figure S4**

**Supplementary Figure S4: Outcome according to *BAALC/ABL1* and *MN1/ABL1* copy numbers at diagnosis in AML patients receiving HSCT in first complete remission (“outcome set”, n=131). (A)** Event Free Survival and **(B)** Overall Survival according to *BAALC/ABL1* copy numbers and **(C)** Event Free Survival and **(D)** Overall Survival according to *MN1/ABL1* copy numbers.

**Supplementary Figure S5**

**Supplementary Figure S5: Landmark analysis for the first 139 days after diagnosis according to *BAALC/ABL1* and *MN1/ABL1* copy numbers at diagnosis in patients receiving chemotherapy (“outcome set”, n=77). (A)** Event Free Survival and **(B)** Overall Survival according to *BAALC/ABL1* copy numbers and **(C)** Event Free Survival and **(D)** Overall Survival according to *MN1/ABL1* copy numbers (n=76).

**Supplementary Figure S6**

**Supplementary Figure S6: Outcome according to *BAALC/ABL1* and *MN1/ABL1* copy numbers at diagnosis in AML patients consolidated with an allogeneic stem cell transplantation in CR/CRi (A)** Cumulative Incidence of Relapse and **(B)** Non-relapse Mortality according to *BAALC/ABL1* copy numbers and **(C)** Cumulative Incidence of Relapse and **(D)** Non-relapse Mortality according to *MN1/ABL1* copy numbers.

**Supplementary Figure S7**

**Supplementary Figure S7: Outcome according to pre-HSCT *BAALC/ABL1* copy numbers prior to allogeneic HSCT. (A)** Event Free Survival and **(B)** Overall Survival in all patients with data available (n=76). **(C)** Event Free Survival and **(D)** Overall Survival patients with low *BAALC/ABL1* copy numbers at diagnosis (n=34) and **(E)** Event Free Survival and **(F)** Overall Survival in patients with high *BAALC/ABL1* copy numbers at diagnosis (n=43).

**Supplementary Figure S8**

**Supplementary Figure S8: Outcome according to pre-HSCT *MN1/ABL1* copy numbers prior to allogeneic HSCT. (A)** Event Free Survival and **(B)** Overall Survival in all patients with data available (n=76). **(C)** Event Free Survival and **(D)** Overall Survival patients with low *MN1/ABL1* copy numbers at diagnosis (n=33) and **(E)** Event Free Survival and **(F)** Overall Survival in patients with high *MN1/ABL1* copy numbers at diagnosis (n=43).

**Supplementary Figure S9**


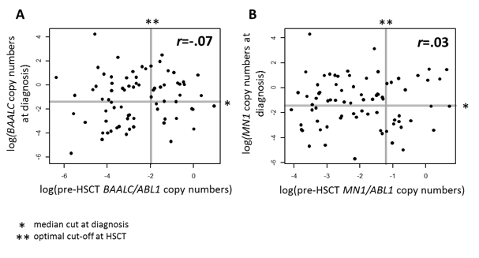


**Supplementary Figure S9: Correlation between copy numbers at diagnosis (in bone marrow) and before allogeneic HSCT (in peripheral blood) (Pearson correlation coefficient) (A)** *BAALC* (n=77) and **(B)** *MN1* (n=76)*.*

**Supplementary Figure 10**

*
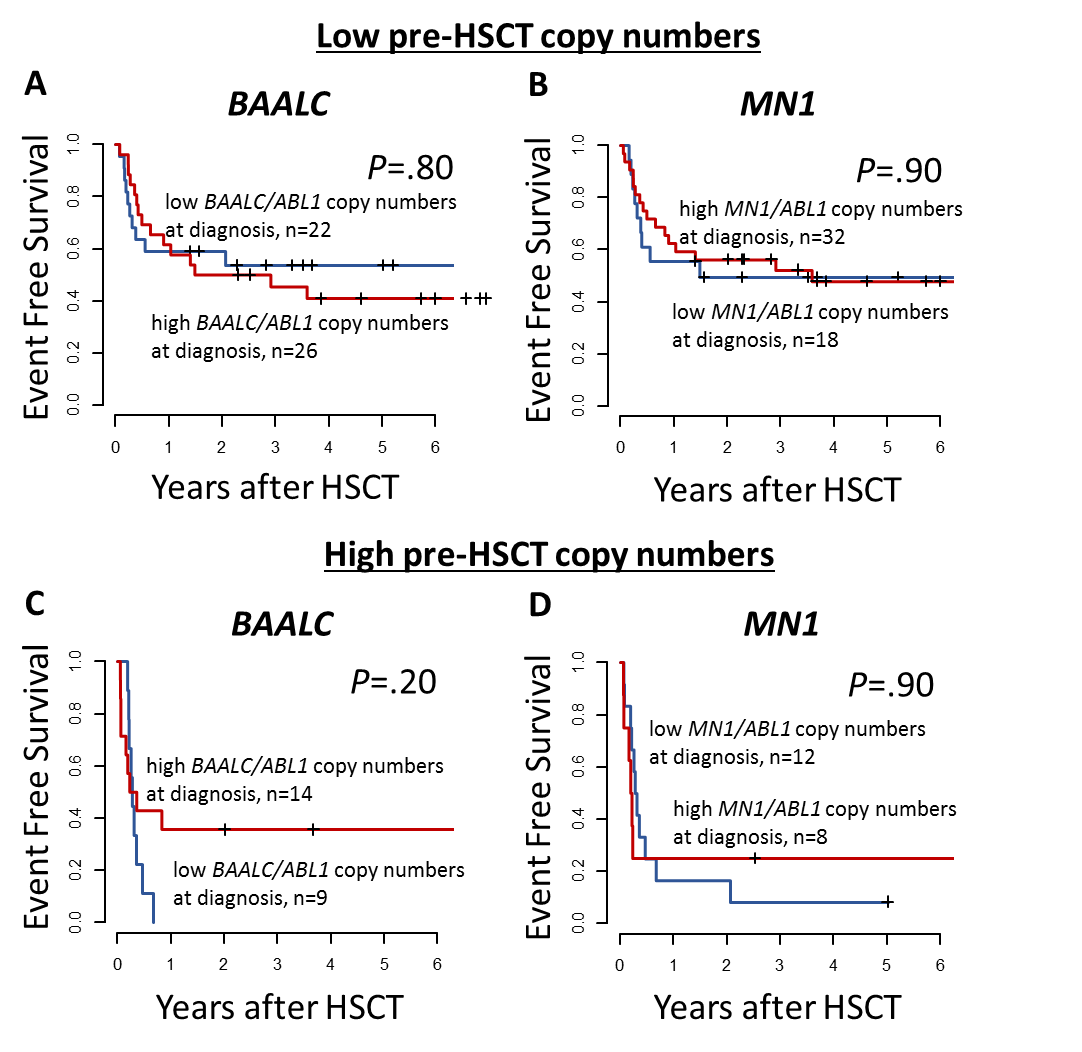
*

**Supplementary Figure S10: Event Free Survival according to diagnostic *BAALC/ABL1* and *MN1/ABL1* copy numbers within AML patients with (A, B)** low copy numbers pre-HSCT or **(C,D)** high copy numbers pre-HSCT**.**
